# Material Characterisation and Computational Thermal Modelling of Electron Beam Powder Bed Fusion Additive Manufacturing of Ti2448 Titanium Alloy

**DOI:** 10.3390/ma14237359

**Published:** 2021-11-30

**Authors:** Qiushuang Wang, Wenyou Zhang, Shujun Li, Mingming Tong, Wentao Hou, Hao Wang, Yulin Hao, Noel M. Harrison, Rui Yang

**Affiliations:** 1 Institute of Metal Research, Chinese Academy of Sciences, Shenyang 110016, China; qswang18b@imr.ac.cn (Q.W.); wthou@imr.ac.cn (W.H.); haowang@imr.ac.cn (H.W.); ryang@imr.ac.cn (R.Y.); 2 School of Materials Science and Engineering, University of Science and Technology of China, Shenyang 110016, China; 3 I-Form, SFI Research Centre for Advanced Manufacturing, Ireland; W.ZHANG2@nuigalway.ie (W.Z.); noel.harrison@nuigalway.ie (N.M.H.); 4 Mechanical Engineering, School of Engineering, College of Science and Engineering, NUI Galway, H91 TK33 Galway, Ireland; 5 Ryan Institute for Environmental, Marine and Energy Research, NUI Galway, H91 TK33 Galway, Ireland

**Keywords:** electron beam powder bed fusion, thermal process modelling, Ti2448 alloy, phase transformation, computational modelling

## Abstract

Ti-24Nb-4Zr-8Sn (Ti2448) is a metastable β-type titanium alloy developed for biomedical applications. In this work, cylindrical samples of Ti2448 alloy have been successfully manufactured by using the electron beam powder bed fusion (PBF-EB) technique. The thermal history and microstructure of manufactured samples are characterised using computational and experimental methods. To analyse the influence of thermal history on the microstructure of materials, the thermal process of PBF-EB has been computationally predicted using the layer-by-layer modelling method. The microstructure of the Ti2448 alloy mainly includes *β* phase and a small amount of α″ phase. By comparing the experimental results of material microstructure with the computational modelling results of material thermal history, it can be seen that aging time and aging temperature lead to the variation of α″ phase content in manufactured samples. The computational modelling proves to be an effective tool that can help experimentalists to understand the influence of macroscopic processes on material microstructural evolution and hence potentially optimise the process parameters of PBF-EB to eliminate or otherwise modify such microstructural gradients.

## 1. Introduction

Powder Bed Fusion (PBF) is a category of Additive Manufacturing (AM) technology that constructs 3D parts in a layer-by-layer manner. The heat source can either be electron beam (PBF-EB, with a common trademark being electron beam melting (EBM)) or laser beam (PBF-LB, with a common trademark being selective laser melting (SLM)) [1]. PBF-EB employs a high energy electron beam as the heat source to heat and melt the powder material, which in turn solidifies rapidly as the electron beam moves on to form the final solid parts. The complex thermal processes during PBF-EB have significant influence on the overall properties and in-service performance of manufactured parts [2]. For example, PBF-EB manufactured components can have a unique microstructure which consists of coarse columnar grains aligned in the <001> orientation and equiaxed grains near the base plate [3]. Gradient microstructure is more likely to be formed in the PBF-EB process due to the extreme solidification conditions, such as high cooling rate, high thermal gradient and intense partitioning behaviour of alloying elements [4]. It is reported that the size of precipitates changes gradually in Inconel 718 alloy through PBF-EB process, which can be used to optimise the mechanical behaviour of the alloy [5]. The PBF-EB 316L stainless steel also shows a hierarchical microstructure. By optimising the process parameters, PBF-EB 316L stainless steel exhibited better mechanical properties and density than the same material manufactured by using powder metallurgy process (hot isostatic pressing) [6].

Mechanical properties of manufactured metallic parts using PBF-EB are closely related to the material microstructures. In order to be able to manufacture PBF-EB products that have desirable mechanical properties, it is necessary to understand the process-microstructure relationship. To date, two different types of computational modelling techniques have been widely applied for computationally predicting the thermal processes of PBF-EB: the moving heat source method [7,8] and the layer-by-layer method [9,10]. The moving heat source method directly models a high energy electron beam spot scanning the powder bed layer along the designed scanning paths (e.g., as presented in [11]). The layer-by-layer approach applies a uniformly distributed heat source instantaneously to a whole layer of the part, which is followed by cooling (as well as solidification) of the whole layer of the part and loose powder material (if included in the model). The moving heat source method is computationally intensive and therefore is generally employed for the computation of a small volume of material, or a single layer build [11]. The layer-by-layer method is relatively computationally economical and therefore is suitable for the thermo-mechanical modelling at the macroscale. Williams et al. [12] predicted the distortion of a cuboid part during PBF-LB by using the layer-by-layer method and the simulation result was within 10% of experimentally measured part distortion. Zhang et al. [13] employed a layer-scaling technique in the computational prediction of temperature and residual stress by using the layer-by-layer method for a PBF-LB process for Ti-6Al-4V alloy. Prabhakar et al. [9] investigated the mechanical behaviour of Inconel 718 during PBF-EB process by using the layer-by-layer method, and the modelling results were validated by using related experimental characterisation.

Ti-24Nb-4Zr-8Sn (Ti2448) alloy is a nontoxic *β*-type titanium alloy. It has relatively good compatibility with the natural bones due to its low elastic modulus in conjunction with a variety of outstanding material properties such as high fatigue strength, considerable ductility and good corrosion resistance [14,15] etc. AM technology has been used to successfully manufacture Ti2448 medical products, such as hip stems, acetabulum cups and interbody fusion cages [16]. Hernandez et al. indicated that columnar *β* grains and α″-martensite plates were observed in the PBF-EB Ti2448 solid part [17]. Yang et al. [18] showed that columnar *β* grains were surrounded by equiaxed *β* grains near the boundary of melt pool in the PBF-LB Ti2448 alloy. The hard-wraps-soft effect caused by this kind of microstructure can improve the mechanical properties of titanium alloy [18]. Liu et al. found that the microstructure of scaffolds manufactured by PBF-EB and PBF-LB were composed of different phases due to the difference in powder bed temperature, and the single β phase of PBF-LB Ti2448 alloy resulted in higher compressive strength and lower Young’s modulus [19]. It can be seen that the microstructure characteristics of PBF-LB and PBF-EB Ti2448 alloy are different and can influence the mechanical behaviours of manufactured parts. In order to obtain components with excellent comprehensive properties, it is necessary to fully understand the influence of process parameters on the microstructure formation by using computational modelling and experiments.

In this study, to analyze the temperature evolution of Ti2448 and its influence on microstructure during PBF-EB, the layer-by-layer approach was employed to computationally predict the thermal process of the PBF-EB AM of Ti2448 alloy at the scale of the overall additively manufactured component. The microstructure of manufactured PBF-EB Ti2448 parts was characterised using experimental methods and compared with the computational modelling results. It is the first time that experimental research on PBF-LB of Ti2448 alloy in conjunction with computational thermal modelling has been investigated. The computational modelling results are used in this paper to facilitate the discussion and interpretation of the experimental and microscopy results of manufactured samples.

## 2. Materials and Methods

### 2.1. Computational Modelling Methods

In former papers from the authors [13,22], a multi-physics computational model for PBF-LB was presented. It coupled a thermal sub-model with a sub-model of solid mechanics, to computationally predict the thermomechanical responses of Ti6Al4V during AM for the purpose of particularly predicting the residual stress. Following mesh convergence and layer scaling factor checks, the model was used to investigate how process parameters of PBF (e.g., scanning strategy) can affect the thermomechanical processes during PBF-LB. Separately, their thermomechanical model was validated using synchrotron radiation on PBF-LB manufactured Ti6AL4V samples to experimentally characterise the residual stress [22]. For the purpose of model validation, multiple Ti6Al4V samples were practically manufactured by using PBF-LB with six different scanning strategies. The residual stress of samples was experimentally measured by using X-ray diffraction testing at a synchrotron radiation facility in Europe. For the same six different scanning strategies, the thermomechanical model was used to computationally predict the residual stress of related Ti6Al4V samples. A reasonable agreement was found between experimental results and computational modelling results of the residual stress of the samples. It proved that the thermomechanical model was reliable and effective, in terms of such as thermal modelling and mechanical modelling. Related details of the model validation process can be found in Zhang [22].

The following changes were made to the above thermomechanical model for PBF-LB of Ti6Al4V in this study for the PBF-EB of Ti2448 alloy.

- The solid mechanics sub-model was deactivated (only the thermal model was active).
- Material properties of Ti2448 were added as defined in Table 1.
- PBF-EB process parameters replaced PBF-LB process parameters (particularly considering the pre-heating step for each layer of powder bed during PBF-EB).

#### 2.1.1. Setup of the Thermal Modelling Process and Simulation Domain

The practical PBF-EB process consists of the following four steps [8,23]:

(a) A thin layer of powders (e.g., layer height of 40 μm) is deposited over a build platform and then is preheated by an electron beam operated in powder pre-heat mode.
(b) A finely focused electron beam (e.g., spot diameter of 200 μm) in melt mode rapidly melts the powders in a localised manner. It rapidly moves around the powder layer, tracing out the required 2D cross section of the 3D metallic parts.
(c) After the fabrication of one layer, the build platform moves down by one layer thickness and is ready for a new layer of loose powder to be spread.

The above three steps are repeated until the 3D component is completed.

For the convenience of the computational modelling in this study, the PBF-EB AM process is simplified to include the following four steps in the computation:

- Preheating step in which the heat source preheats the powders to below melt temperature for a period of time defined as the preheating step time, *t*_p_, which represents the time spent on scanning the part by using the electron beam during the preheating step.
- Preheating ‘cooling’ step represents the time when the electron beam leaves the part and moves to preheat other areas of the powder bed for a period of preheating ‘cooling’ step time, *t*_c_. The total preheating time, *t*_l_, is the sum of *t*_c_ and *t*_p_.
- Active layer melting step is when the finely focused electron beam operates in melt mode, heating the powders to above liquidus temperature. By active layer, it refers to the newly added layer of powders. The active layer melting step time, *t*_m_, can be calculated as the electron beam spot diameter divided by the electron beam moving speed for melting the powders, and assumes a constant beam scanning speed within a layer.
- Melting cooling step is the step in which the electron beam is deactivated and the roller/coater spreads a new thin layer of powders on the powder bed.

The above four computational steps (i-iv) are repeated for each layer during the PBF-EB thermal modelling process. After finishing the final layer, there is an additional final cooling step in which the part is allowed to cool to room temperature for 8 h 43 min with no more deposition of powders and no more heat input in the computational modelling.

**Figure 1:**
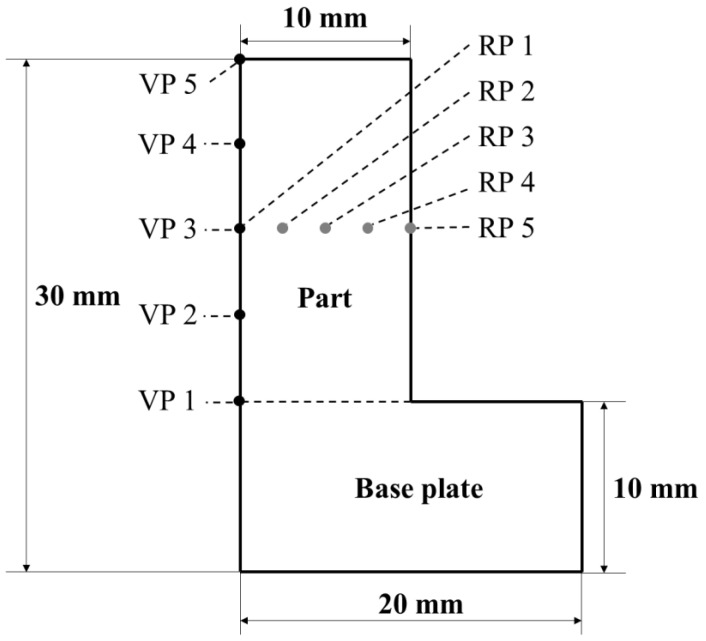
Geometry of the simulation domain and position of sampling points (“VP” for sampling points along the centreline i.e., height of cylindrical sample, “RP” for sampling points along the radius of cylindrical sample at its mid-height).

During the practical PBF-EB process, a cylinder sample in the dimension of 20 mm diameter × 20 mm height was built on a 10 mm thick base plate. As shown in Figure 1, a 2D axisymmetric simulation domain was employed in the thermal process modelling. In the practical PBF-EB process, the base plate temperature was approximately constant (around 773 K). In the computational model, the priority is the thermal history of the part, rather than the whole base plate. For the convenience of computational modelling, isothermal boundary condition (constant temperature of 773 K) was applied at the interface between the part and the base plate. Therefore, the size of the base plate that was employed in the setup of computational modelling (20 mm × 10 mm as shown in Figure 1) does not have to reflect the size of actual base plate. A python-based script for finite element software ABAQUS FEA package (Dassault Systemes, Johnston, SC, USA, 2017) was programmed to convert the macroscale geometry into 286 layer-by-layer slices [13,24]. During the computation for each layer, an element-birth technique [13,21,25] was employed. The converged (less than 0.19% nodal temperature difference) mesh size of 23 μm × 23 μm was employed for the part. The four-node linear axisymmetric heat transfer quadrilateral mesh type of DCAX4 was employed.

The results of thermal modelling were analysed at 10 different sampling points, including five evenly distributed sampling points (the VP points) along the height of the 2D part at its centre and five sampling points (the RP points) along the radius of the 2D part at its mid height as shown in Figure 1. The 5 VP points correspond to layer 1, layer 72, layer 143, layer 214 and layer 285 in the modelling, respectively.

#### 2.1.2. Thermal Modelling for the PBF-EB Process by Using Finite Element Method

The thermal modelling in this study was implemented by computationally solving the following governing equations using the finite element method with ABAQUS. The energy equation that was employed in the thermal modelling for the PBF-EB process is [10,26]:

(1) $$\rho_{s}C_{p}\frac{dT}{dt}+\rho_{s}\frac{d\left(fL\right)}{dt}+\nabla \cdot q=0$$

where $\rho_{s}$ is for density, $C_{p}$ for specific heat capacity, *T* for temperature, *t* for time, *L* for latent heat of fusion, and q for heat flux. The liquid fraction *f* is assumed to be a linear function of temperature [27] as:

(2) $$f=\{\begin{array}{c} 0\;T<T_{S}\\ \frac{T-T_{S}}{T_{L}-T_{S}}\;T_{S}\leq T\;\leq \;T_{L}\\ 1\;T>T_{L} \end{array}$$

where $T_{S}$ and $T_{L}$ are the solidus and liquidus temperature, respectively.

In the layer-by-layer modelling, all finite elements of a layer were subjected to simultaneous material deposition and energy input processes [13]. The uniformly distributed volumetric heat sources for both preheating step and active layer melting step were applied for each whole active layer.

In the preheating step, the volumetric power density at the active layer of the 2D part in the layer-by-layer process is calculated as:

(3) $$Q_{v}=\frac{AP_{p}}{v_{p\;}t_{p}d_{m}H}$$

where *A* is the heat source absorption coefficient, $P_{p}$ is the electron beam power for preheating, $v_{p\;}$ is the electron beam scanning speed during the preheating process, $t_{p}$ is the preheating step time, $d_{m}$ is the melt pool depth and *H* is the hatch spacing. The electron beam power for preheating $P_{p}$ is calculated as [23]:

(4) $$P_{p}=UI_{p}$$

where U and $I_{p}$ are the acceleration voltage and electron beam current for preheating, respectively. The preheating step time $t_{p}$ can be calculated as:

(5) $$t_{p}=\frac{A_{p}}{A_{b}}t_{l}$$

where $A_{p}$ is the cross-sectional area of the part in question, $A_{b}$ is the surface area of the entire powder bed and $t_{l}$ is the overall preheating time (28 s) for each layer during the manufacturing process. Equation (1) was computationally solved for $t_{p}$ seconds for the preheating process.

In the preheating ‘cooling’ step of the thermal modelling, the preheating cooling step time $t_{c}$ can be calculated as:

(6) $$t_{c}=t_{l}-t_{p}$$

Equation (1) was computationally solved for $t_{c}$ seconds for the preheating ‘cooling’ process.

In the active layer melting step of the thermal modelling, the volume power density that was applied at the top layer of the computational domain is calculated as [13]:

(7) $$Q_{v}=\frac{AP_{m}}{d_{s}d_{m}H}$$

where $P_{m}$ is the electron beam power for melting and $d_{s}$ is the electron beam diameter for melting. The electron beam power for melting $P_{m}$ is calculated by:

(8) $$P_{m}=UI_{m}$$

where $I_{m}$ is the electron beam current for melting. The melting step time $t_{m}$ in the thermal modelling is calculated as [20]:

(9) $$t_{m}=\frac{d_{s}}{v_{m}}$$

where $v_{m}$ is the electron beam scanning speed during the material melting process. Equation (1) was computationally solved for $t_{m}$ seconds for the active layer remelting process.

The computational melting cooling step time for each active layer is 10.45 s, corresponding to the practical PBF-EB manufacturing process.

The above four processes (i.e., preheating step, preheating ‘cooling’ step, active layer melting step and melting cooling step) were repeated in the computational modelling until the end of the PBF-EB process, since which moment the volumetric heating source at the active layer of the simulation domain was set to be equal to zero (i.e., electron beam was turned off). Equation (1) was solved until the 2D part cooled to room temperature.

#### 2.1.3. Thermal Boundary Conditions

Axisymmetric boundary condition was applied on the left boundary of the simulation domain (as shown in Figure 1). The solid base plate was isothermally kept at 773 K in the thermal modelling as well as in the practical PBF-EB trials.

The following heat losses were considered in the thermal modelling: heat conduction between the active layer (i.e., the newly added layer of powders) and the solidified material of the previous layer or the base plate, heat radiation at the top surface of the active layer to the chamber. The heat transfer between the powder bed and the side of the solidified part was dealt with in a way similar to heat convection, and the related details can be found in the authors’ previous papers [13,24,25]. Due to the fact that the PBF-EB process is normally conducted in a vacuum environment, the heat convection at the top surface of the active layer was not considered in the modelling. The heat flux due to conduction can be formulated as [28]:

(10) q=−k∇T

where k is the temperature-dependent thermal conductivity of the material. The heat radiation at the top surface of the active layer was considered in the modelling before the next layer was added [13]:

(11) $$q_{\mathrm{rad}}=\varepsilon \;\sigma \left(T_{s}{}^{4}-T_{r}{}^{4}\right)$$

where $q_{\mathrm{rad}}$ is the heat flux due to active layer radiation, *ε* is the emissivity, *σ* is the Stefan-Boltzmann constant, $T_{s}$ is the surface temperature of the part and $T_{r}$ is the build chamber temperature [29]. Here, the emissivity of the active layer surface *ε* and the Stephan-Boltzmann’s constant *σ* were set as 0.35 [30] and 5.669 × 10^−8^ W/(m^2^·K^4^) [31], respectively.

The heat flux between the 2D part at its side surface and the surrounding powder bed [13,24] can be formulated as:

(12) $$q_{\mathrm{conv}}=h\left(T_{s}-T_{r}\right)$$

where *h* is the heat transfer coefficient [30].

#### 2.1.4. Material Properties and Process Parameters

For the PBF-EB thermal modelling, the material of both the part and the base plate was defined as Ti2448 to avoid thermal expansion mismatch [32]. The temperature dependent Ti2448 material properties that were employed in the computation were assumed to vary linearly with temperature between the values that are presented in Table 1. The thermal conductivity and specific heat were detected by Flashline^TM^-5000 Thermal Properties Analyzer according to GB/T 22588-2008. The PBF-EB process parameters were found from Arcam EBM User Mannual (Arcam AB, 2011, Mölndal, Sweden), as shown in Table 2.

**Table 1:** Ti2448 material properties.

Temperature (K)	Thermal Expansion (10^−5^/K)	Thermal Conductivity (W/m^2^/K)	Specific Heat (J/kg/K)
333	0.87 [33]		
370		8.35	478
873		15.8	445
983	1.06 [33]		
1273		19	470
1928	1.35 [33]		

### 2.2. Experimental Methods

#### 2.2.1. PBF-EB Printing Process

**Figure 2:**
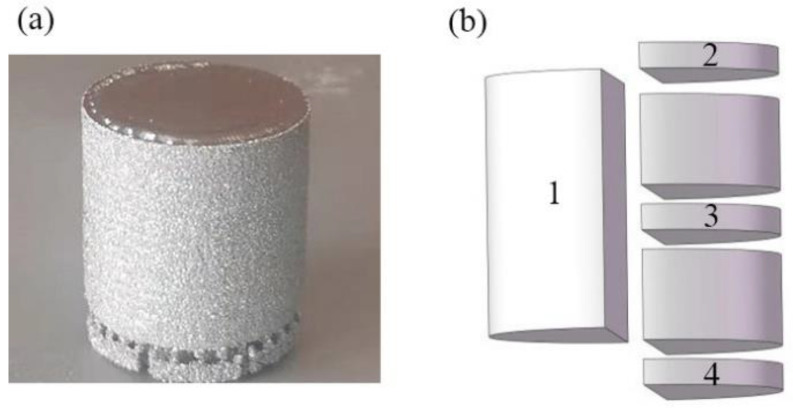
(**a**) Ti2448 sample manufactured by PBF-EB. (**b**) Sketch illustrating how the sample was sectioned for metallographic analysis. (Position-2, 3 and 4 is 18 mm, 10 mm and 2 mm from the base plate, respectively).

Arcam A1 PBF-EB system was used to fabricate dense samples in this work with a 0.5 mm accuracy. The pre-alloyed powder (particle diameter ranges from 45 to 106 µm) used in PBF-EB process was produced by using argon atomization of a Ti2448 ingot. The chemical composition of the powder was (wt.%) Nb-24.43, Zr-3.93, Sn-8.22, O-0.22 and Ti-bal. The CAD model of samples with a size of φ20 × 20 mm was designed using Magics 17.0 (Materialise, Leuven, Belgium) software, and then the model was sliced and imported into PBF-EB equipment for parameters setting. The printing started after the vacuum in the build chamber and electron gun became under 5.0 × 10^−4^ mbar and 5.0 × 10^−6^ mbar, respectively. The electron beam preheated the base plate with a dimension of 170 × 170 × 10 mm to 773 K for 20 min, and then the equipment started to spread the powders for printing with a layer thickness of 70 µm. The electron beam moved by tracing out the designed geometry to melt the powders in the active layer of the powder bed and then the base plate got lowered by one layer thickness. These processes were repeated until the completion of the part. It was noted that the printing time of each layer was influenced by the melting area, and the printing procedure ended after 8 h. When the base plate cooled to room temperature, the non-melted powder could be removed by compressed air to obtain the as-fabricated sample (Figure 2a).

#### 2.2.2. Material Characterization Methods

The samples were sectioned by using wire electric discharge machining (EDM) as shown in Figure 2b. The metallographic specimens were made at positions 1–4, respectively, so as to characterise the material microstructure on the cross-sections of the sample and separately on a centre plane of the sample. The microstructural features of the prepared samples were analysed by using ZEISS-AXIO optical microscope (OM), MIRA3 TESCAN scanning electron microscope (SEM) and Talos transmission electron microscope (TEM). Metallographic samples for OM and SEM observations were etched in a reagent (2 vol% HF, 6 vol% HNO_3_ and 92 vol% H_2_O) for 15 s until the crystal morphology could be seen clearly. Backscattered electron imaging (BSE) was used to examine mechanically polished samples without corrosion. The TEM samples were processed by twin-jet electro-polishing (Tenupol-5), and the electrolyte was made of 6% HClO_4_, 59% CH_3_OH and 35% CH_3_(CH_2_)_3_OH in volume percent. The grain size in different locations of the sample was measured by electron back scatter diffraction (EBSD) with a step size of 6 μm, and the size of the detection area was 2.1 × 2.3 mm. The indexing rate of all the EBSD samples was higher than 95%. The EBSD sample was electropolished in the electrolyte mentioned above. The data of grain size was exported from Channel 5 software with the grain boundary definition of 10°. To characterise the phases of material at positions 2–4 of the as-fabricated sample, X-ray diffraction (XRD, D8 DISCOVER with Cu Kα radiation) measurement was conducted with a scanning angle range of 20°–90°.

### 2.3. Computation of Phase Equilibrium

The equilibrium phase diagram of Ti2448 was calculated with the PANDAT 2020 (CompuTherm LLC, Middleton, MA, USA) package in association with the Ti-Nb-Zr-Sn-O thermal and mobility database covering the temperature range of 273 K–1273 K. The two basic phases of Ti2448, i.e., α and β, were considered in the computation of phase equilibrium.

## 3. Results

### 3.1. Thermal Modelling Results

#### 3.1.1. Temperature Profile along the Height of the Manufactured Part

**Figure 3:**
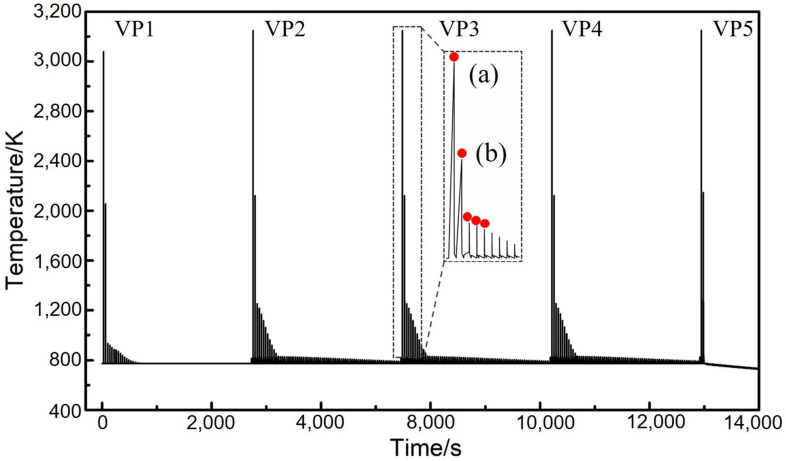
Evolution of temperature at the five vertical sampling points (VP1, VP2, VP3, VP4 and VP5) with time. (Zone (**a**) illustrates the thermal history of the active layer during melting and cooling, and zone (**b**) illustrates the influence of the subsequent processes on the temperature history of the current layer).

In the thermal modelling, the part was virtually sliced into 286 layers and each layer was deposited on top of the previous layer step by step. The thermal modelling was implemented for 11 h 46.28 min of the PBF-EB process, including 3 h 3.28 min of the additive manufacturing process and the post-manufacturing cooling process of 8 h 43 min. Temperature histories of the five vertical sampling points (VP1, VP2, VP3, VP4 and VP5, shown in Figure 1) are illustrated in Figure 3. In this figure, zone (a) illustrates the temperature of the active layer in question at the end of the corresponding melting process (step 3 of the computational modelling process). The maximum temperature was above 3200 K, corresponding to the first peak of temperature for each curve (e.g., the curve corresponding to VP3). The deposition, preheating, melting and cooling processes of the subsequent layers of material caused periodic oscillation of temperature at the vertical sampling point in question. The influence of subsequent processes on the temperature history of the current layer (or vertical sampling point) in question can be found in zone (b).

Figure 3 indicates that the temperature at the five vertical sampling points changed with time as the part was built layer upon layer. In order to enhance the visibility of the figure, the results are only illustrated up to 12,000 s of the PBF-EB process. With the deposition of sequential layers, the maximum temperatures at VP1 was always the lowest compared with the maximum temperature at the other four vertical sampling points, while the five vertical sampling points share similar temperature history (Figure 3). Overall, temperature increases from the bottom towards the top of the manufactured part during the PBF-EB process. The first deposition layer has the lowest temperature, because of the significant heat conduction to the base plate. With the sequential deposition of material, during the layer-by-layer PBF-EB process, the thermal influence of the base plate on the newly laid layer of material gradually becomes less significant. Therefore, the temperature temporal evolution near the mid height of the part (e.g., VP2, VP3 and VP4) becomes relatively unaffected by the height of the vertical sampling points, and the corresponding three curves in Figure 3 are similar. This agrees with the findings in other PBF-EB process studies [34]. The temperature temporal evolution at VP5 is relatively different from that of other four vertical sampling points, as no further layers were added after 3 h 3.28 min. The temperature at VP5 only oscillates for two cycles, and it quickly starts to monotonically decrease. After finishing the manufacturing of the part, the part was eventually cooled to room temperature during the post-printing cooling process.

**Figure 4:**
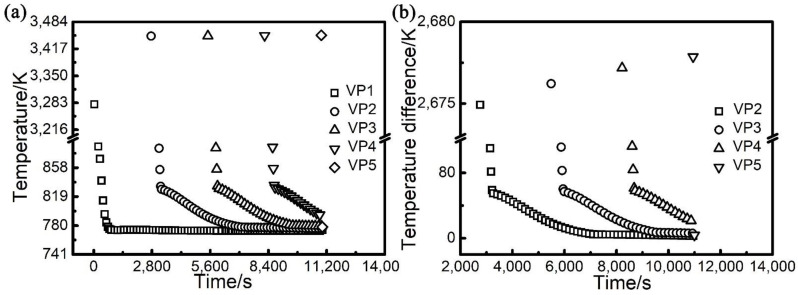
Evolution of peak temperature (**a**) and temperature difference (**b**) with time at different points along the building direction of the studied sample shown in Figure 1.

In order to characterise the temperature variation of the part in the vertical direction during the PBF-EB process, the peak temperature of each thermal cycle was chosen to make the peak temperature variation curve of VP1-VP5 sampling points with time, as shown by the red dot in Figure 3a,b. It can be seen that there exists very rapid periodic oscillation of material in temperature during the AM process due to the layer-by-layer approach. In order to make the temporal evolution of temperature more visible, the peak temperature of each thermal cycle and how it evolves with time at respective vertical sampling points is shown in Figure 4a. It can be seen that the peak temperature increases gradually from VP1 to VP4. Moreover, the temperature difference (TD) is defined, which is the peak temperature at the sampling points of VP2, VP3, VP4 and VP5 (Figure 1) of the part minus the peak temperature at VP1. The evolution of TD with time at respective vertical sampling points is shown in Figure 4b. It can be seen that the TD values are all positive, which further confirms that the temperature of material near the base plate is the lowest in the overall part. The temporal evolutions of the TD at VP2, VP3 and VP4 are very similar to one another. At VP2, for example, during the deposition, preheating, melting and cooling of the active layer, the TD decreased from approximately 2675 K to ~1 K. Such a process is not monotonic but significantly oscillates up and down because the deposition, preheating, melting and cooling of the PBF-EB is repetitive. Overall, it can be seen that the value of TD at VP4 is higher than VP3, and that at VP3 is higher than VP2. This further confirms an increasing temperature profile of the part along its height.

**Figure 5:**
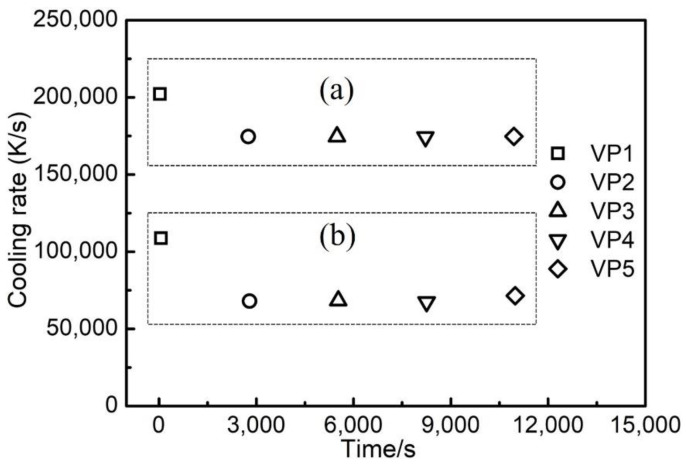
Maximum cooling rate at the five vertical sampling points: (**a**) at the end of the melting process of the layer in question, (**b**) resulting from the thermal processes of subsequent layers.

The cooling rate at the five vertical sampling points (Figure 1) was calculated based on the temperature history that is shown in Figure 3. Figure 5a illustrates the maximum cooling rate at respective vertical sampling points up to the end of the melting step (as shown in Figure 3a). Figure 5b illustrates the maximum cooling rate at the vertical sampling points, which results from the influence of the subsequent layers (as shown in Figure 3b). It can be seen that the highest level of cooling rate is in the order of magnitude of 10^5^ K/s. It is close to the practically measured results of 10^5^~10^6^ K/s in other work [35,36]. Figure 5a indicates that the peak cooling rate occurred immediately after the completion of the melting step (Figure 3a) of the active layer. During the process of the subsequent layers (Figure 3b), the cooling rate at the vertical sampling points in question significantly decreased. It can be seen, as shown in Figure 5, that the cooling rate at the sampling point VP1 is the highest. It is higher than the cooling rate at VP2, VP3, VP4 and VP5 by approximately 3 × 10^4^ K/s. This is due to the fact that the isothermal base plate took the role of a powerful heat sink that very effectively extracted heat by conduction from the part at its bottom during the PBF-EB process. A similar decrease in the cooling rate along the building direction of alloy 718 is found in other work [37].

#### 3.1.2. Temperature Profile in the Radial Direction of the Manufactured Part

**Figure 6:**
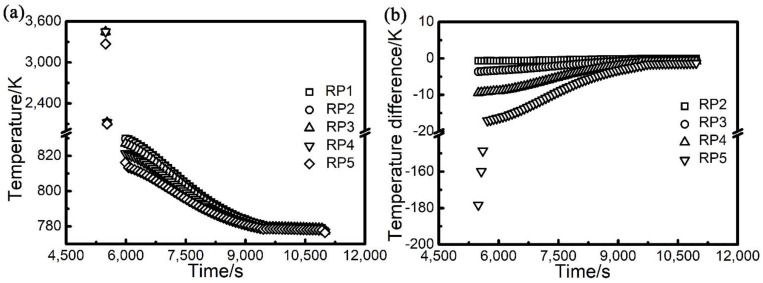
Evolution of peak temperature (**a**) and temperature difference (**b**) with time at different radial sampling points along the radial direction of the studied sample shown in Figure 1.

The peak temperature of the material at radial sampling points (RP1 to RP5) of the manufactured part reflects the material temperature distribution along the radial direction. Their evolution with time along the VP3 layer is shown in Figure 6a. It can be seen that the peak temperature decreases from RP1 to RP5 on the VP3 layer. The TD in the radial direction of the manufactured part is defined to be the temperature at the four radial sampling points RP2, RP3, RP4, RP5 minus the temperature at the sampling point RP1 (as shown in Figure 1), at the mid-height of the manufactured part. Its evolution with time is shown in Figure 6b. It can be seen that the value of TD is negative, which means that the temperatures at RP2, RP3, RP4 and RP5 was lower than that at RP1 during the PBF-EB process. TD has the highest absolute value at RP5. Overall, the temperature near the surface of the part is lower than that near the centreline of the part, and there is a decreasing profile of temperature along the radius of the part. This phenomenon was caused by the heat loss of the part to the surrounding powder bed at the side surface of the part [13,22]. The absolute value of TD in the radial direction decreases with time at all the sampling points (RP2 to RP5), which is due to the heat conduction process in the part tends to make the temperature profile uniform, as increasingly more materials are deposited above the mid-height of the part.

**Figure 7:**
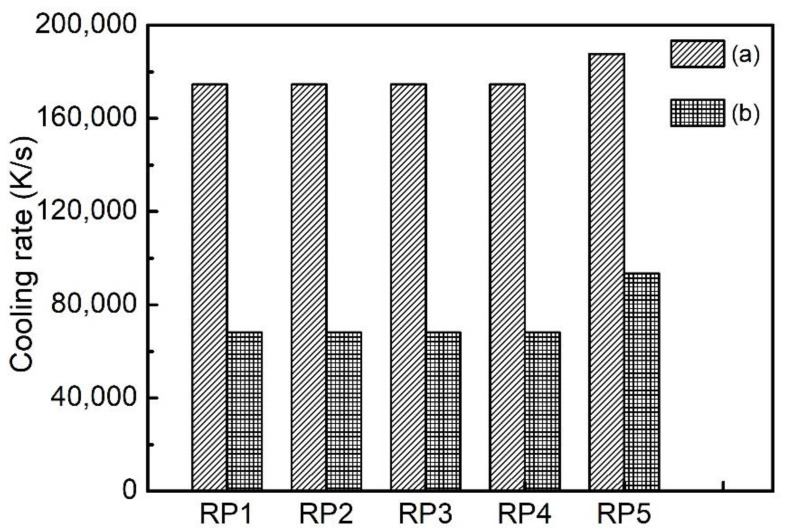
Maximum cooling rate at the five radial sampling points: (**a**) at the end of the melting process of the layer in question, (**b**) resulting from the thermal processes of subsequent layers.

The maximum cooling rate at the five radial sampling points RP1, RP2, RP3, RP4 and RP5 of the VP3 layer is shown in Figure 7. During the processes of material deposition, preheating and melting of the layer at the mid height of the part, the highest cooling rate was in the order of magnitude of 10^5^ K/s. It can be seen in Figure 7, the surface of the part (i.e., at RP5) has the highest level of cooling rate, which is higher than the cooling rate at RP1, RP2, RP3 and RP4 by approximately 1 × 10^4^ K/s. As the subsequent layers were deposited on the part above its mid height, the cooling rate at the sampling points RP1, RP2, RP3 and RP4 significantly decreased to the order of magnitude of 7 × 10^4^ K/s, while the cooling rate at RP5 decreased to the order of magnitude of 9 × 10^4^ K/s (Figure 7b).

#### 3.1.3. Thermal Process during the Natural Cooling Process Post Manufacturing

**Figure 8:**
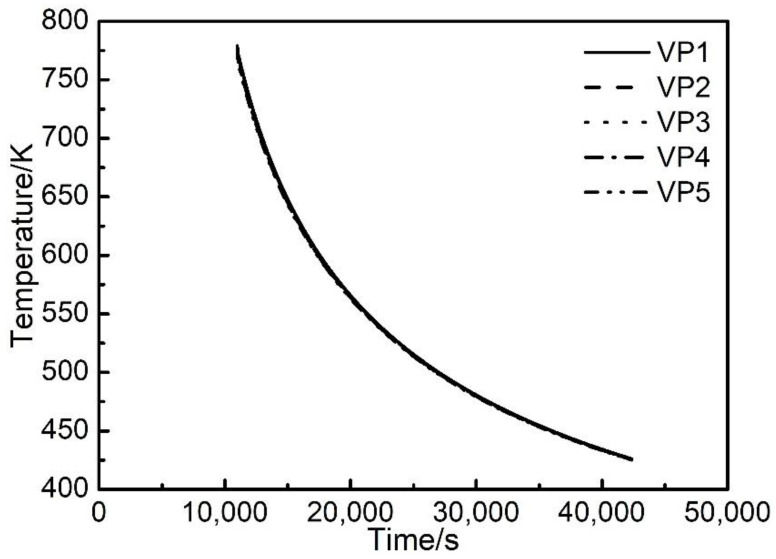
Evolution of temperature of the part at the five vertical sampling points with time during natural cooling process post manufacturing.

In the thermal modelling results that were analysed in Section 3.1.1 and Section 3.1.2, the data analysis was focused on the manufacturing process, i.e., the step No. 1, 2, 3, 4 of the physical PBF-EB process. After the manufacturing process was completed, the manufactured part was left in the build chamber to cool for a period of time before it could be taken out. Figure 8 illustrates the thermal modelling results of the temperature evolution of the part at the five vertical sampling positions with time during the natural cooling process post manufacturing. It can be seen that at vertical sampling point VP1 in this figure, the material temperature continuously decreased from 774 K to 426 K between 3 h 3.28 min and 11 h 46.28 min. The temperature evolutions at the five different vertical sampling points are very close to one another, and the maximum difference in temperature is 7.3 K. The temperature at the top surface of the part is lower than that at the bottom of the part. In the radial direction of the sample, the temperature profile turns out to be relatively uniform. The maximum difference in temperature between the five radial sampling positions is 0.87 K.

### 3.2. Material Characterization Results

**Figure 9:**
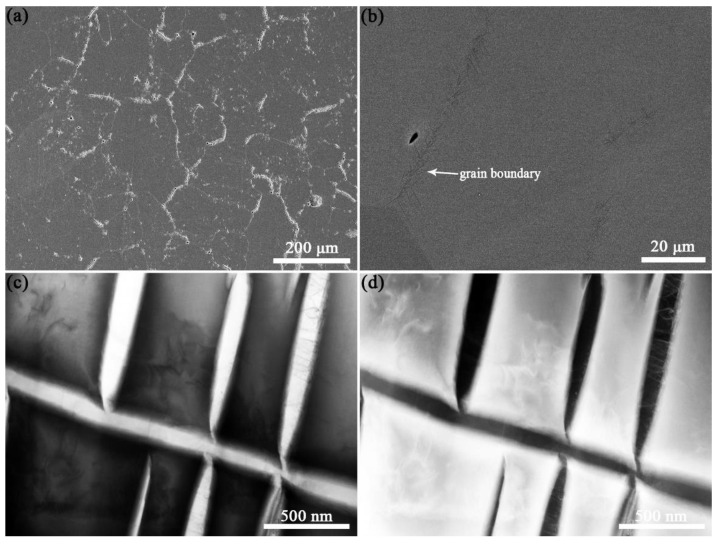
SEM image (**a**), BSE image (**b**), Bright-Field STEM image (**c**) and High-Angle Annular Dark Field image (**d**) of the cross-section of the sample. The BSE image (**b**) and High-Angle Annular Dark Field image (**d**) indicates that the composition of precipitates is different from that of matrix.

**Figure 10:**
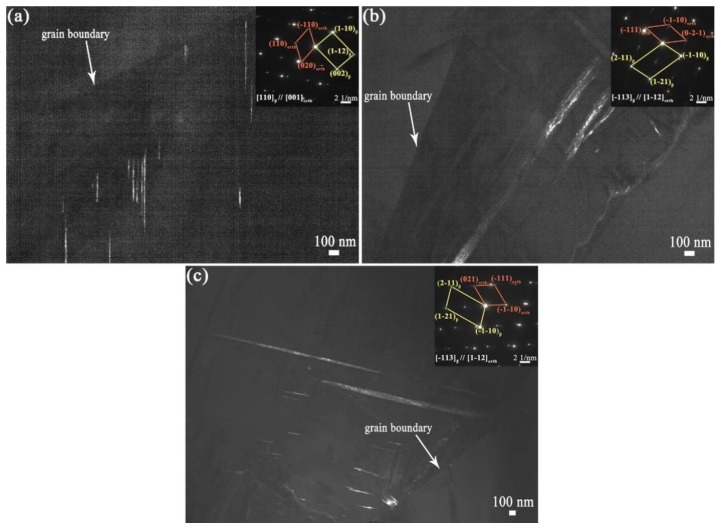
Dark-Field TEM image and Selected-Area Diffraction image of Position 2 (**a**), Position 3 (**b**), Position 4 (**c**) shown in Figure 2b.

**Figure 11:**
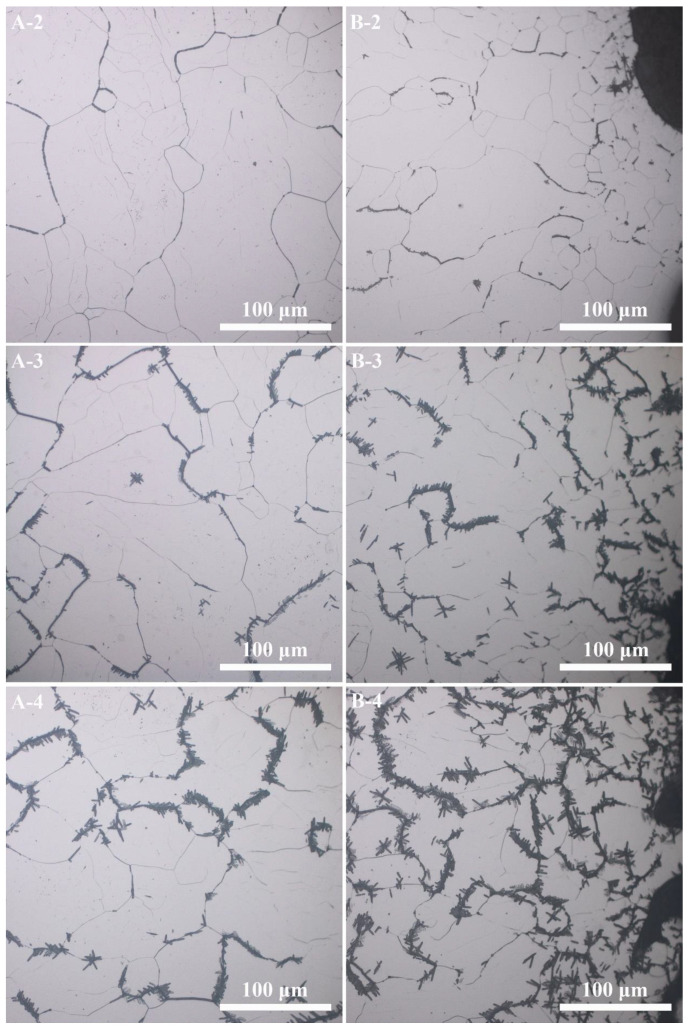
Optical image of the cross-section in different segments of the sample. (The pictures with the letter (“**A**”) represents the central area of the cross-section, and the pictures with the letter (“**B**”) represents the cross-section of sample near its side surface. Number 2–4 on the pictures represents segment 2–4 as illustrated in Figure 2b).

**Figure 12:**
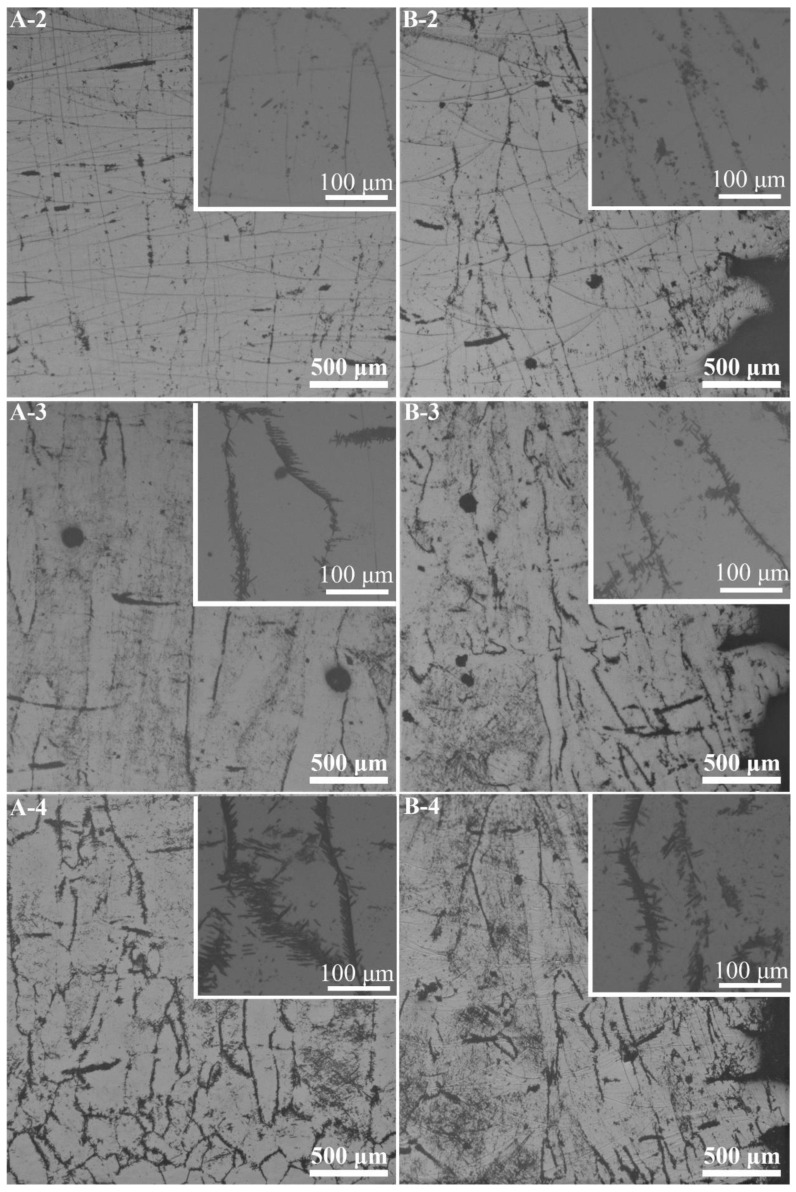
Optical image of vertical section in different segments of sample. (The pictures with the letter (“**A**”) represents the central area of the vertical section, and the pictures with the letter (“**B**”) represents the vertical section of the sample near its surface. Numbers 2–4 on the pictures represent the top, middle, bottom segments on the vertical section, respectively, as shown in Figure 2b).

Figure 9a,b show the microstructure characteristics of the PBF-EB produced Ti2448 sample. The PBF-EB sample displays a microstructure with coarse columnar grains and fine lath phases. There are a small amount of fine lath phases distributed on the grain boundaries and inside the grains according to the SEM and BSE images of the cross-section of the sample. Comparing the Bright-Field image (Figure 9c) with the High-Angle Annular Dark Field image (Figure 9d), it is found that the composition of precipitates is different from that of the matrix. It is verified by TEM observation that the fine lath phase is an orthorhombic structure precipitated from β columnar grains (Figure 10). The precipitates at the grain boundaries and in the grains have the same structure. Comparing the microstructure of material at the cross-section and vertical section (Figure 11, Figure 12), it can be seen that coarse columnar β grains tend to form parallel to the building direction, and a layer of equiaxed grains is distributed near the baseplate. Such a microstructure is the inherent feature of the additive manufacturing process and is consistent with the results elsewhere [38]. Observing the distribution trend of the precipitates at the positions 2–4 (Figure 2b), it can be seen that the content of fine lath phase decreases from the bottom to the top along the building direction of the sample, and only very limited precipitates can be seen on the top of the part (Figure 11A-2). In addition, it can be seen that more fine lath phase exists near the side surface of the sample than that around the centre line of the sample at the same height (Figure 11B-2 and A-2). It can be seen in Table 3 that the *β* grain size increases slightly along the building direction of the sample. Meanwhile, the *β* grain size near the centre line of the sample is larger than that near the side surface of the sample.

**Figure 13:**
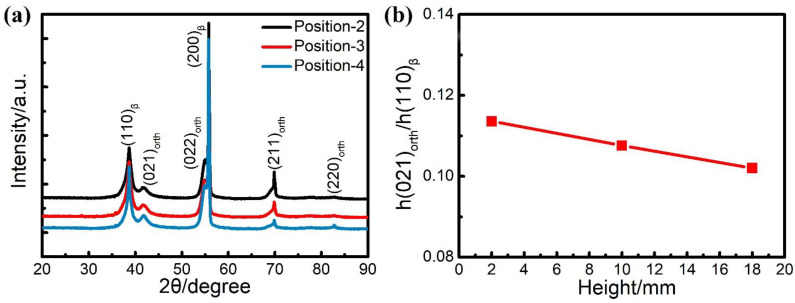
XRD analysis results of PBF-EB Ti2448 alloy at different heights. (**a**) XRD diffraction patterns of Ti2448 sample at positions 2–4 shown in Figure 2b. (**b**) The change of height ratios of diffraction peak (021)_orth_ to (110)_β_ along the height direction.

By analysing the XRD diffraction patterns of the sample at positions 2–4 as illustrated in Figure 2b and excluding a few heteropeaks caused by experimental artefacts, it can be concluded that there are α” phase and *β* phase in the Ti2448 alloy manufactured by PBF-EB (Figure 13a). Given that X-ray diffraction analysis shows that the studied sample has a similar texture at different positions along height direction, the ratio of peak height of the α” phases with respect to the β phase is used to describe the variation of volume fractions of the α” phases along the height of the sample. It can be seen that the height ratios of (021)orth to (110)β peaks decreases with the increase of sample height (Figure 13b). This phenomenon indicates that the content of the α” phase decreases with the increase of sample height [39], which is consistent with the distribution law of microstructure pictures.

## 4. Discussion

### 4.1. Variation of the α” Phase

Metastable *β*-titanium alloys generally exhibit a single *β* phase at room temperature. In the isothermal aging process, the transitional phase (the α” phase or ω phase) will precipitate from the supersaturated *β* phase, and it will gradually evolve into equilibrium α phase with the extension of aging time [40,41]. For the isothermal ω phase, it forms by solute diffusion i.e., it rejects *β*-stabilising elements into the matrix during aging and the collapse of two of every three (222)_β_ planes in the [111]_β_ direction into a single plane [42]. The ω precipitates were found to be strongly deficient in Nb and weakly deficient in Zr for the Ti-24Nb-[0–8]Zr (at.%) alloys [43]. The ω phase can provide the dominant nucleation position for α phase, which is conducive to the precipitation of α phase [44]. The solute redistribution occurs at the very beginning of the *β* decomposition process, resulting in the composition fluctuation on the matrix. The *β*_stabilizer_ lean region is unstable and easy to transform into an orthorhombic structure by shear and shuffle mechanisms [45]. During aging, the α” phase was observed to evolve toward a hexagonal α structure [46]. For the equilibrium α phase, the phase transition path can be described as the shear of the {112} [47] plane along the [111] direction accompanied by the shuffling of the cross atomic layers and the uniform contraction along the specific crystal direction [48]. The formation of α phase is a diffusion- displacement type phase transition, which is similar to the formation of ω phase and α” phase. The α phase can precipitate directly from *β* matrix or be transformed from a transition phase [49]. The morphology and distribution of α phase are related to the heat treatment or processing conditions [50,51].

For hot-rolled Ti2448 alloy, the main transformation path during aging is *β* phase (bcc)→α” phase (orth)→α phase (hcp). There are equiaxed Nb-rich and Nb-lean regions with an average size of 2–3 nm in hot-rolled Ti2448 alloy [52,53]. In the process of phase transformation, the Nb-lean region first transforms into α” phase whose structure is close to the *β* matrix (bcc), then the structure of α” phase continuously evolves towards hcp via orthorhombic structure when the solute diffusion and lattice distortion proceed simultaneously. Finally, the *β*→α phase transformation is completed [54]. It is reported that the α” phase precipitates from *β* matrix of Ti2448 alloy during aging at 648–848 K [55].

**Figure 14:**
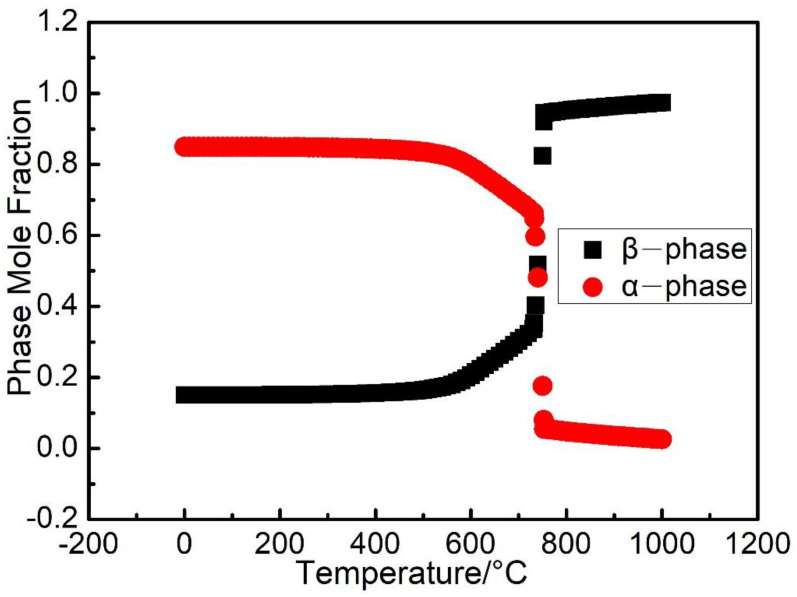
The calculated equilibrium phase diagram of Ti-24Nb-4Zr-7.85Sn-0.22O (wt.%) alloy.

**Figure 15:**
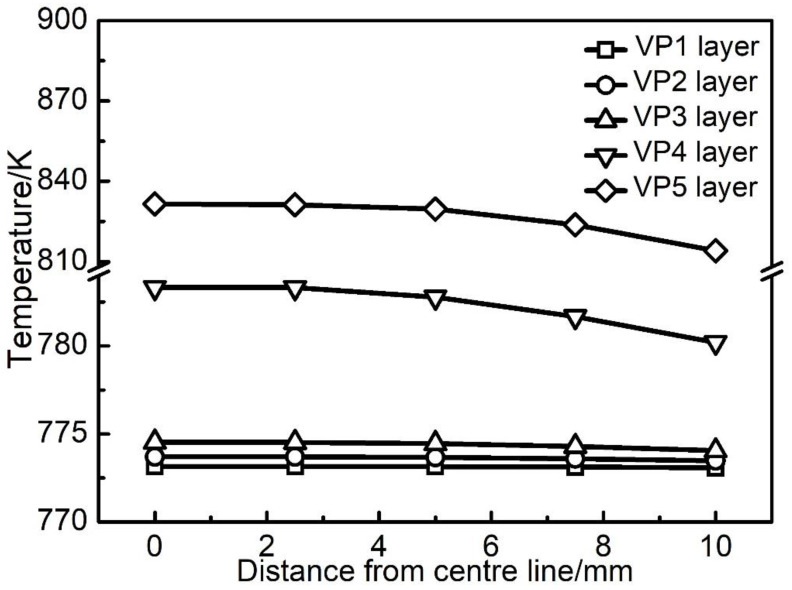
Temperature profile along the radial direction of VP1-VP5 layers of the sample shown in Figure 1 at 10,986.9 s (when the melting process of the sample ends).

The calculated thermal history of the studied sample (Figure 3, Figure 4 and Figure 6) shows that the temperature of the sample is kept in the range of 648 K to 848 K during the most time of the PBF-EB process, which is also the temperature range of α” phase precipitation in Ti2448 alloy [39]. This may facilitate the α” phase precipitation in the studied samples, as confirmed by the XRD (Figure 13) results shown in Section 3. It is interesting that the content of α” phase decreases along the building direction and increases along the radial direction (Figure 11). The reasons for the variation of α” phase content can be summarized as follows: Firstly, aging time can significantly affect the content of precipitates. According to Figure 3, the temperatures of the sampling points VP1-VP4 are approximately stable around the level of 773 K after several cycles of drastic temperature fluctuations. This stage is similar to the aging process, which is conducive to the precipitation of α” phase. The longer the aging time at the precipitation temperature range of α” phase, the more α” phase will precipitate. In the PBF-EB process, the aging time of the sampling points in the temperature range of 773-848 K decreases from VP1 to VP4 in the building direction (Figure 3), which is the main reason for the content of α” phase decreases along the building direction. Secondly, aging temperature is also an important factor affecting the content of precipitates. The calculated equilibrium phase diagram of Ti-24Nb-4Zr-7.85Sn-0.22O (wt.%) alloy is shown in Figure 14. It can be seen from this figure that the content of α phase decreases with the increase of temperature before the transus point, while that of *β* phase shows an opposite trend. Given that the α” phase is the intermediate phase of *β*→α transformation, the change of α” phase content with temperature is similar to that of α phase. Therefore, the positive temperature gradient of VP1-VP5 (Figure 4 and Figure 15) and negative temperature gradient of RP1-5 (Figure 6 and Figure 15) caused the decreasing profile of α” phase in the building direction and increasing profile of α” phase in the radial direction, respectively.

### 4.2. Variation of β Grain Size

It is reported that the equiaxed grain layer near the substrate is formed by heterogeneous nucleation due to co-melting and alloying of deposited powders material close to the base plate [56]. PBF-EB process can result in a high cooling rate and great thermal gradient on the solidification front. The thermal gradient is conducive to grain growth along the preferred crystal direction, and it also promotes the formation of epitaxy between layers, resulting in unidirectional columnar grain growth in additively manufactured parts [38]. In addition, the composition of liquid and solid are similar during PBF-EB, as a result, the wettability of the liquid phase at the surface of the solid phase of the same alloy during PBF-EB is excellent and the wetting angle approaches 0° [57]. This means no nucleation barrier upon remelting and solidifying of the previous layer is present, so the columnar *β* grains can elongate through multiple layers. The reasons for the variation of grain size are summarized as follows: Firstly, the temperature is a main factor affecting grain growth. It is known that the aging temperature has more effect than the aging time on grain size. Figure 15 shows the temperature profiles along the building direction and the radial direction at 10986.9 s, when the last layer of the sample has been melted and before the completion of the manufacturing process. The temperature at the top of the sample is 58 K higher than that at the bottom, which is conducive to the rapid growth of grains, making the grain size at the top of the sample slightly larger than that at the bottom (Table 3). Due to the decreasing temperature profile along the radial direction (Figure 6), the *β* grains near the sample surface are smaller than those near the sample centre line. Secondly, the cooling rate is another mechanism affecting *β* grain size. A high cooling rate is not beneficial to grain growth [58]. As shown in Figure 7, the cooling rate at the side surface of the sample is significantly higher than that at the centre of the sample on the same height. The grains near the centre of the sample are easier to grow than the grains at the side surface, so the grain size has a decreasing profile in the radial direction of the sample. Besides, the partially melted powder at the contact interface between the part and the powder bed provides a large number of heterogeneous nucleation points, which is helpful to reduce the grain size [4,56]. This paper only discusses the influence of thermal history on the variation trend of precipitates content and grain size.

## 5. Conclusions

In this study, the thermal process of PBF-EB was computationally predicted by using the layer-by-layer modelling method. The microstructure of PBF-EB-Ti2448 alloy was characterized by OM, SEM, TEM and XRD, which mainly consists of α” phase and small amount of *β* phase. The computational modelling results of temperature histories, temperature differences and cooling rates along the height and radius of a cylinder part have revealed the driving factors behind the experimentally observed profiles of α” phase fraction and *β* grain size in multiple directions. Overall, due to heat conduction between the consolidated part and the surrounding powder bed as well as heat radiation, material temperature is higher at the top of the part compared with that the bottom of the part. The temperature at the side surface of the cylindrical part is lower than that near the centreline of the part. The highest cooling rate is at the bottom of the part and the side surface of the part. Such thermal processes of PBF-EB and the corresponding temperature profile of part during AM result in different aging times and different temperatures at different sections of a manufactured sample. They caused a gradient along sample height and a gradient along the sample radius in α phase content and in grain size. This work could be further developed into an integrated through-process thermal history based microstructural evolution model, similar to recent computational PBF-LB [59,60] and weld [61,62] simulation tools. Such tools can be used by experimentalists and in industry to optimise the design of PBF-EB process parameters to achieve optimal material microstructures in order to benefit in-service mechanical performance.

## Figures and Tables

**Table 2:** EBM process parameters.

Symbol	Modelling Parameters	Unit	Value
*U*	Acceleration voltage	V	60000
*I*_p_	Current for preheating	mA	14.6
*I*_m_	Current for melting	mA	8.5
*v*_m_	Scanning speed for melting	mm/s	1704.3
*v*_p_	Preheating scanning speed	mm/s	10000
*T*_p_	Substrate preheat temperature	K	773
*A*	Heat source absorption coefficient		0.9 [34]
*d*_s_	Heat source spot diameter for melting	μm	200
*d*_m_	Melt pool depth	μm	150
*H*	Hatch spacing	μm	200
	Powder layer thickness	μm	70

**Table 3:** *β* Grain size (µm) at different segments on the sample.

Position Along the Height of Sample	Around Sample Centre Line	Around Sample Side Surface
Top	111.0 ± 59.2	90.8 ± 67.1
Middle	65.3 ± 30.9	57.2 ± 29.1
Bottom	52.2 ± 25.4	48.3 ± 24.6

(The lognormal distribution function was used to fit the data, and the average value and standard deviation were taken as the grain size data at different positions of the sample, as shown in Figure 11.)

## Data Availability

Data sharing is not applicable for this article.

## Nomenclature

$\rho_{s}$	Density of solid, kg/m^3^
$C_{p}$	Specific heat capacity, J/(kg·K)
*L*	Latent heat of fusion, J/kg
$Q_{v}$	Energy density, W/m^3^
*A*	Heat source absorption coefficient
$P_{p}$	Electron beam power for preheating, W
*H*	Hatch spacing, m
U	Acceleration voltage, V
$P_{m}$	Electron beam power for melting, W
$d_{m}$	Melt pool depth, m
$d_{s}$	Heat source spot diameter, m
k	Thermal conductivity of solid, W/(m^2^·K)
∇T	Temperature gradient, K/m
*q*	Heat flux, W/m^2^
$q_{\mathrm{rad}}$	Radiation heat flux, W/m^2^
*ε*	Emissivity coefficient
*σ*	Stefan-Boltzmann constant, W/(m^2^·K^4^)
